# Gastroprotective Effect of Selenium on Ethanol-Induced Gastric Damage in Rats

**DOI:** 10.3390/ijms13055740

**Published:** 2012-05-11

**Authors:** Jeong-Hwan Kim, Shin-Hyung Park, Soo-Wan Nam, Yung-Hyun Choi

**Affiliations:** 1 Department of Biomaterial Control (BK21 Program), Dong-Eui University Graduate School, Busan 614-714, Korea; E-Mails: 12845@deu.ac.kr (J.-H.K.); swnam@deu.ac.kr (S.-W.N.); 2 Department of Pathology, College of Oriental Medicine, Dong-Eui University, Busan 614-052, Korea; E-Mail: psyji043@hanmail.net; 3 Department of Biotechnology and Bioengineering, Dong-Eui University, Busan 614-714, Korea; 4 Blue-Bio Industry RIC, Dong-Eui University, Busan 614-714, Korea; 5 Anti-Aging Research Center, Dong-Eui University, Busan 614-714, Korea; 6 Department of Biochemistry, College of Oriental Medicine, Dong-Eui University, Busan 614-052, Korea

**Keywords:** selenium, ethanol, gastric mucosal lesions, radical scavenging enzymes

## Abstract

In the present study, we examined the gastroprotective effect of selenium against ethanol-induced gastric mucosal lesions in rats. The gastric mucosal lesions were produced by oral administration with various concentrations of ethanol for three days, and 80% ethanol treatment was determined to be the optimal condition for induction of gastric damage. To identify the protective effect of selenium on ethanol-induced gastric damage, various doses of selenium were given as pretreatment for three days, and then gastric damage was induced by 80% ethanol treatment. Selenium showed a protective effect against ethanol-induced gastric mucosal lesions in a dose dependent manner. Specifically, 100 μg/kg selenium showed the highest level of gastroprotection. In addition, selenium markedly attenuated ethanol-induced lipid peroxidation in gastric mucosa and increased activities of radical scavenging enzymes, such as superoxide dismutase (SOD), catalase, and glutathione peroxidase in a dose-dependent manner. Histological data showed that 100 μg/kg selenium distinctly reduced the depth and severity of the ethanol induced gastric lesion. These results clearly demonstrate that selenium inhibits the formation of ethanol-induced gastric mucosal lesions through prevention of lipid peroxidation and activation of enzymatic radical scavenging.

## 1. Introduction

The gastric mucosa is one of the most important tissues in an organism, because of its function, structure, and the pathological processes that can take place in this tissue [1,2]. Generally, an imbalance between aggressive chemical agents *versus* protective substances, such as superoxide dismutase (SOD), catalase, and glutathione peroxidase, causes acute gastric mucosal lesions [3,4]. Ethanol is an ulcerogenic agent that is known to produce erosions, ulcerative lesions, and petechial bleeding in the mucosa of the stomach [5–7]. Ethanol rapidly penetrates the gastric mucosa, causing membrane damage, exfoliation of cells, erosion, and ulcer formation [8,9]. Ethanol-induced gastric mucosal lesions in humans are mainly mediated through the release of inflammatory mediators, which induce vasoconstriction/ischemia, oxidative stress, lipid peroxidation and then cell death [10–16]. In experimental animals, the oral administration of ethanol rapidly induces gastric mucosal lesions, and these ethanol-induced lesions are commonly used to study both the pathogenesis and therapy of human ulcerative disease [17–19].

Selenium, an essential dietary element for mammals, has important metabolic functions in animals, including protection of membrane lipids and macromolecules from oxidative damage produced by peroxides [20], and activation of important antioxidant proteins, thioredoxin reductase and several selenoproteins [21,22]. In addition, neuroprotective effects of selenium have been reported at the experimental level, in both methamphetamine and 6-hydroxydopamine-induced toxicities [23], as well as at the clinical level, with positive responses to therapy with selenium being shown in neurodegenerative diseases [24–26]. However, its biological and pharmacological effects are still poorly defined. In particular, there are no reports of the gastroprotective effect of selenium against ethanol-induced gastric mucosal lesions. Because ethanol-induced gastric mucosal lesions are mediated by the generation of free radicals, we hypothesized that selenium can inhibit ethanol-induced gastric mucosal lesions through a protective effect that may directly involve its antioxidant property. Therefore, this study was designed to investigate the gastroprotective effect of selenium by measuring the amount of lipid peroxidation and by comparing the activities of enzymatic scavengers, such as SOD, catalase and glutathione peroxidase.

## 2. Results and Discussion

### 2.1. Determination of the Optimal Condition for Induction of Ethanol-Induced Gastric Mucosal Lesions in Rats

**Figure 1:**
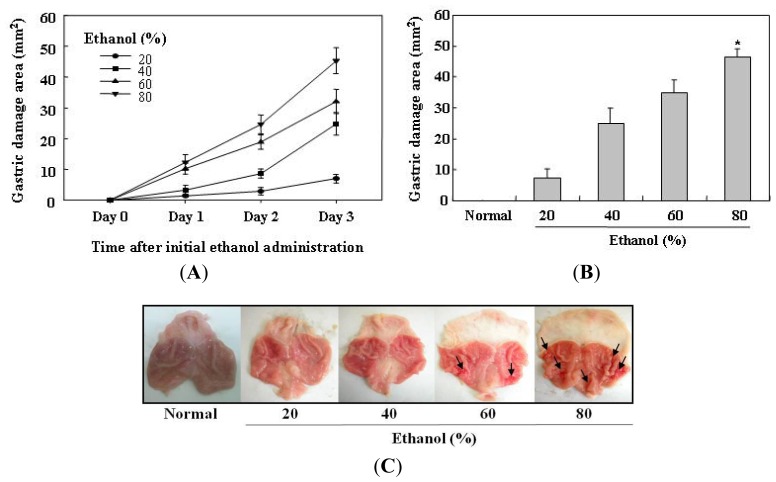
Determination of optimal ethanol-induced gastric mucosal injury model in rats. (**A**) Ulcerogenic effects in rats with various doses of ethanol, when administered over a 3 day period; (**B and C**) Various concentrations (20, 40, 60, and 80%) of ethanol were given by orogastric gavage for 3 days. Gastric damage area (mm^2^) was judged macroscopically by clear depth of penetration into the gastric mucosal surface. 80% ethanol induced the highest increase in the area of gastric damage. The arrows show the gastric mucosal lesions, such as erosions, bleeding, and ulcers. Values are expressed as the mean ± SEM. ^*^ *p* < 0.05, significantly different from the untreated normal rats.

In the present study, gastric mucosal lesions in rats were induced by ethanol, and the lesions produced were studied for identification of the gastroprotective effect of selenium. To find the optimal dose of ethanol for producing gastric mucosal damage, various concentrations (20, 40, 60, and 80%) of ethanol were administered to the rats for 3 days. Gastric lesions were judged macroscopically by the depth of penetration into the gastric mucosal surface. As shown in Figure 1, oral administration of ethanol for 3 days increased the area of gastric damage in all ethanol-treated rats in a dose dependent manner. However, ethanol administered for 1 and 2 days showed a relatively low rate of gastric damage area. In contrast, 80% ethanol given for 3 days produced the largest area of gastric damage. Also, gastric mucosal lesions such as erosions, bleeding, and ulcers were clearly observed in rats receiving 80% ethanol for 3 days. Therefore, 80% ethanol administered for 3 days was determined to be the optimal condition for selenium study.

### 2.2. Selenium Inhibits Ethanol-Induced Gastric Mucosal Lesions in Rats

**Figure 2:**
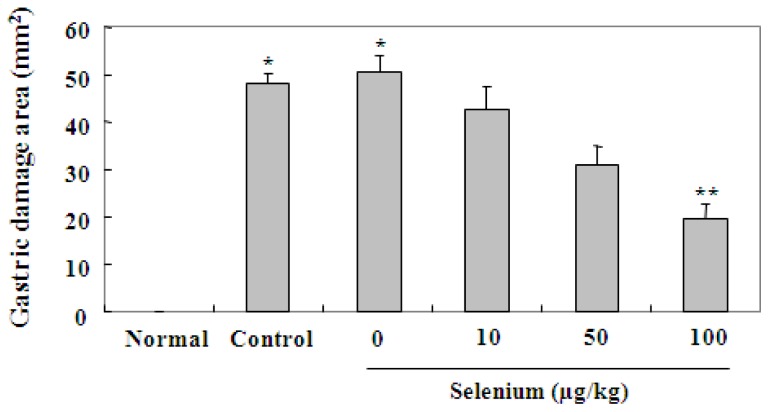
Effect of selenium on the formation of gastric lesions after oral administration of ethanol to rats. A vehicle (selenium, 0 μg/kg) and three doses (10, 50, and 100 μg/kg of body weight) of selenium were given as pretreatment for 3 days, and then gastric mucosal damage was induced by 80% ethanol treatment for 3 days. Selenium pretreatment significantly attenuated the gastric damage area in the stomach in a dose dependent manner, compared with 80% ethanol alone. Values are expressed as the mean ± SEM. ^*^ *p* < 0.05, significantly different from the untreated normal rats; ^**^ *p* < 0.01, significantly different from the control rats.

**Figure 3:**
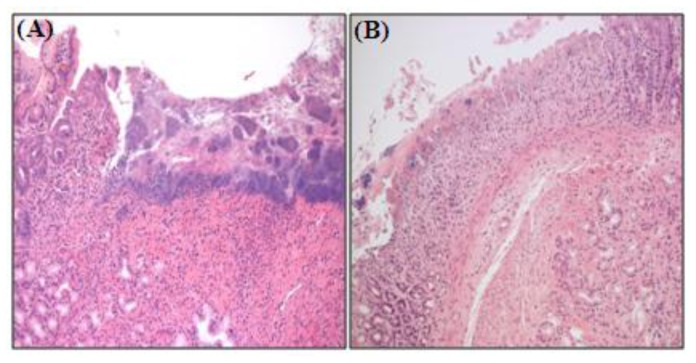
An immediate gastroprotective effect of selenium on ethanol-induced gastric lesions in rats. One hundred μg/kg selenium was given as pretreatment for 3 days, and then gastric mucosal damage was induced by 80% ethanol treatment for 3 days. (**A**) Gastric ulcer in ethanol-treated rat; (**B**) Gastric mucosa in selenium-pretreated rat. 100 μg/kg selenium prevents ethanol-induced gastric lesions by direct protection of gastric mucosa.

As described in the experimental protocol, a vehicle (selenium, 0 μg/kg) and three doses (10, 50, and 100 μg/kg) of selenium were given as pretreatment for 3 days, and then gastric mucosal lesions were induced by 80% ethanol treatment. To confirm the effect of selenium against ethanol-induced gastric mucosal damage, gastric lesions were measured macroscopically by clear depth of penetration into the gastric mucosal surface in all experimental groups. As shown in Figure 2, oral administration of 80% ethanol for 3 days clearly increased the gastric damage area in the stomach, compared with the untreated normal group (^*^ *p* < 0.05). On the other hand, selenium pretreatment significantly attenuated the area of gastric damage in the stomach in a dose dependent manner, compared with 80% ethanol alone (control group). Especially, pretreatment of 100 μg/kg selenium was the most effective in inhibiting ethanol-induced gastric mucosal lesions (^**^ *p* < 0.01). As shown in Figure 3, histological examination clearly showed that 100 μg/kg selenium distinctly reduced the depth and severity of ethanol-induced gastric mucosal lesions. These results show that selenium inhibits ethanol-induced gastric mucosal lesions through protection of gastric mucosa. These results show that selenium acts immediately to protect the gastric mucosa against ethanol-induced damage.

### 2.3. Selenium Inhibits Ethanol-Induced Gastric Mucosal Lesions through Prevention of Lipid Peroxidation and Activation of Radical Scavenging Enzymes

**Figure 4:**
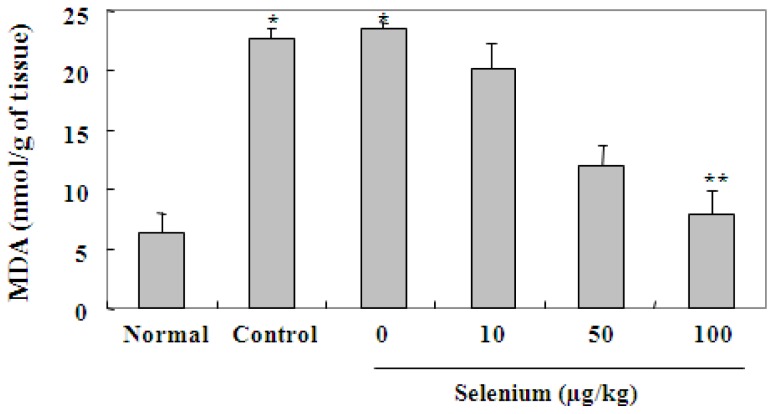
Effect of selenium against lipid peroxidation in gastric mucosa. The rats were pretreated with a vehicle (selenium, 0 μg/kg) and three doses (10, 50, and 100 μg/kg of body weight) of selenium for 3 days, and then gastric mucosal damage was induced by 80% ethanol treatment for 3 days. Malonylaldehyde (MDA) production was estimated by using a thiobarbituric acid reaction. Selenium pretreatment reduced the level of MDA in a dose dependent manner in comparison to the control group. Values are expressed as the mean ± SEM. ^*^ *p* < 0.05, significantly different from the untreated normal rats; ^**^ *p* < 0.01, significantly different from the control rats.

In the gastric mucosa, ethanol causes gastric damage through lipid peroxidation and the generation of free radicals. To evaluate the direct gastroprotective mechanism of selenium against ethanol-induced gastric mucosal lesions, the level of lipid peroxide and the activities of scavenging enzymes were measured in the stomach of all experimental groups. In the case of lipid peroxidation, malonylaldehyde (MDA) production was estimated by using a thiobarbituric acid reaction. As shown in Figure 4, 80% ethanol applied for 3 days in the control and vehicle (selenium, 0 μg/kg) groups significantly increased the level of MDA in gastric tissue in comparison to the untreated normal group (^*^ *p* < 0.05), whereas selenium pretreatment reduced the level of MDA produced in a dose dependent manner in comparison to the control group. Specifically, pretreatment of 100 μg/kg selenium for 3 days showed a significant decrease in the level of MDA (^**^ *p* < 0.01), compared with 80% ethanol alone. These experimental results clearly reveal that selenium inhibits the formation of ethanol-induced gastric mucosal lesions, through prevention of lipid peroxidation.

**Figure 5:**
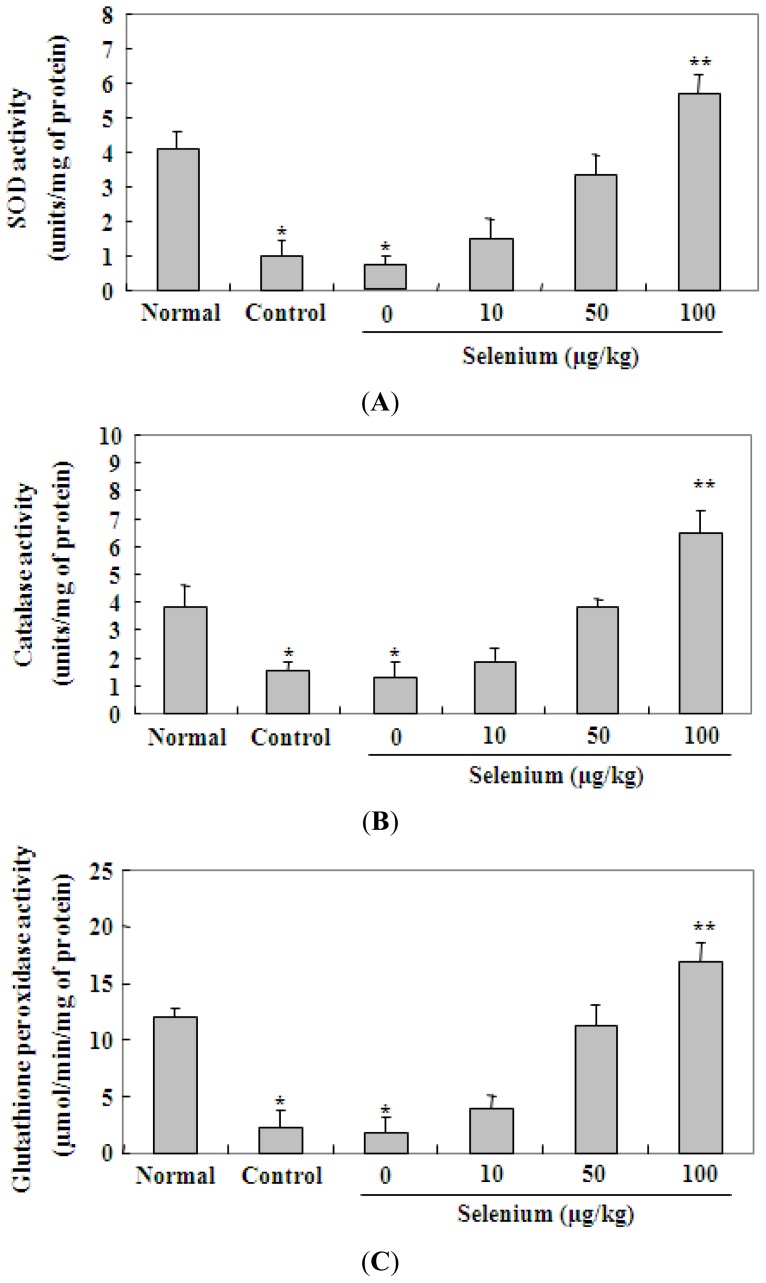
Effect of selenium on activities of radical scavenging enzymes in gastric mucosa. A vehicle (selenium, 0 μg/kg) and three doses (10, 50, and 100 μg/kg of body weight) of selenium were given as pretreatment for 3 days, and then gastric mucosal damage was induced by 80% ethanol treatment for 3 days. Selenium pretreatment increased activities of SOD (**A**), catalase (**B**) and glutathione peroxidase (**C**) in a dose dependent manner in comparison to the control group. Values are expressed as the mean ± SEM. ^*^ *p* < 0.05, significantly different from the untreated normal rats; ^**^ *p* < 0.01, significantly different from the control rats.

We also investigated the effect of selenium on the activities of radical scavenging enzymes, such as SOD, catalase, and glutathione peroxidase in gastric musosa. As shown in Figure 5, 80% ethanol administered for 3 days in the control and vehicle (selenium, 0 μg/kg) groups significantly reduced activities of SOD, catalase and glutathione peroxidase in comparison to the untreated normal group (^*^ *p* < 0.05). However, selenium pretreatment increased the activities of these enzymes in a dose dependent manner in comparison to the control group. In particular, 100 μg/kg selenium applied for 3 days showed a significant (^**^ *p* < 0.01) increase in the activities of the radical scavenging enzymes, compared with 80% ethanol alone.

## 3. Experimental Section

### 3.1. Chemicals

Sodium selenite (Na_2_SeO_3_) as a selenium reagent and absolute ethanol were purchased from Sigma-Aldrich Chemicals (St. Louis, MO., USA). Sodium selenite was dissolved in dimethyl sulfoxide (DMSO) immediately before use and administered intragastrically to rats in a volume of 5 mL/kg. Ethanol was administered by orogastric gavage, with an appropriate feeding needle in a volume of 5 mL/kg. All chemicals were of the highest purity available.

### 3.2. Animals

Male Sprage-Dawley rats (230~250 g, 7 weeks old) were provided by Daehan Biolink Co. (Seoul, Korea). Rats were placed in cages with wire-net floors in a controlled room (temperature 22~24 °C, humidity 70~75%, light on at 6 a.m. and off at 6 p.m.; 12 h light and 12 h dark) and they were fed a normal laboratory diet. Typically, rats were fasted for 18 h before experimental use. Following the first dose of ethanol, rats were provided with food for the remainder of the study. Rats were also allowed tap water throughout the study period. The animal experiment was performed in accordance with guidelines established by the Animal Care and Use Committee of Dong-Eui University and approved by the committee.

### 3.3. Induction and Evaluation of Ethanol-Induced Gastric Lesions

Various concentrations (20, 40, 60, and 80%) of ethanol were given by orogastric gavage for 3 days. Rats were killed under deep ether anesthesia 1 h after the last oral administration of ethanol at various time points (1–3 days). The stomach was opened along the greater curvature, and then rinsed in 0.9% saline. Gastric lesions were counted with the aid of a 7× magnifier and an attached metric scale. The area (in mm^2^) of individual gastric ulcers was also estimated by determining the product of the measured ulcer length and its width. These initial studies were solely designed to help determine an optimal gastric damaging dose as well as the time point for subsequent antiulcer drug testing against ethanol-induced gastric lesions.

### 3.4. Experimental Protocol

To investigate the gastroprotective effect of selenium, rats were divided into six groups (*n* = 8 rats per group). The normal group received only distilled water for 3 days, in comparable volume by the oral route. The control group received only 80% ethanol for 3 days. Each of the remaining four groups was pretreated with a vehicle (selenium, 0 μg/kg) and three doses (10, 50, and 100 μg/kg) of selenium for 3 days, and then gastric lesions were induced by 80% ethanol treatment for 3 days. The concentrations of selenium were selected on the basis of the preliminary results obtained from cytotoxicity studies using a broad concentration range for this reagent. All rats were killed under deep ether anesthesia 1 h after the last oral administration of ethanol. The rat stomachs were promptly excised, weighed, and chilled in ice-cold 0.9% NaCl. After washing with 0.9% NaCl, the mucosa was homogenized in 50 mM potassium phosphate buffer at pH 7.5. Mitochondria and cytosol fractions were prepared according to the method of Hogeboom [27]. The quantity of protein was measured by Bradford protein assay [28].

### 3.5. Malondialdehyde Levels

To identify the inhibitory effect of selenium on the generation of lipid peroxide, lipid peroxidation was determined by measuring malonylaldehyde (MDA) production by using a thiobarbituric acid reaction [29,30]. Briefly, the stomach homogenate was supplemented with 8.1% sodium dodecyl sulfate, 20% acetic acid (pH 3.5), and 0.8% thiobarbituric acid, and boiled at 95 °C for 1 h. After cooling with tap water, the reactants were supplemented with *n*-butanol and pyridine (15:1; v/v), shaken vigorously for 1 min, and centrifuged for 10 min at 3500 × g. Absorbance was measured at 532 nm. The lipid peroxide level was calculated from the standard curve using the MDA tetrabutylammonium salt. MDA concentrations were expressed as nmol/g of tissue.

### 3.6. SOD Assay

To investigate whether selenium affects the activity of radical scavenging enzymes, the activity of SOD in the gastric mucosa was measured according to the method of McCord and Fridovich [31]. The standard assay was performed in 3 mL of 50 mM potassium phosphate buffer at pH 7.8 containing 0.1 mM EDTA in a cuvette thermostated at 25 °C. The reaction mixture contained 0.1 mM ferricytochrome *c*, 0.1 mM xanthine, and sufficient xanthine oxidase to produce a reduction rate of ferricytochrome *c* at 550 nm of 0.025 absorbance unit per min. Tissue homogenate was mixed with the reaction mixture (50 mM potassium phosphate buffer, pH 7.8 containing 0.1 mM EDTA, 0.1 mM ferricytochrome *c*, and 0.1 mM xanthine). Kinetic spectrophotometric analysis was started, with the addition of xanthine oxidase at 550 nm. Under these conditions, the amount of SOD required to inhibit the reduction rate of cytochrome *c* by 50% was defined as 1 unit of activity. The results were expressed as units/mg of protein.

### 3.7. Catalase Assay

To exam whether selenium affects the activity of radical scavenging enzymes, the activity of catalase in the gastric mucosa was measured according to the method of Aebi [32]. The standard assay was performed in 3 mL of 50 mM potassium phosphate buffer at pH 7.0 (1.9 mL) containing 10 mM H_2_O_2_ (1 mL) and tissue homogenate (100 μL). Under these conditions, the amount of catalase required to decompose 1.0 μmol of H_2_O_2_ per min at pH 7.0 at 25 °C was defined as 1 unit of activity. Absorbance was measured at 240 nm for 2 min, and the results were expressed as units/mg of protein.

### 3.8. Glutathione Peroxidase Assay

To demonstrate whether selenium affects the activity of radical scavenging enzymes involved in gastroprotection, the activity of glutathione peroxidase in the gastric mucosa of rats was determined by a modified method of Lawrence and Burk [33]. The reaction mixture consisted of glutathione peroxidase assay buffer (50 mM potassium phosphate buffer pH 8.0, 0.5 mM EDTA) and NADPH assay reagent (5 mM NADPH, 42 mM reduced glutathione, and 10 units/mL glutathione reductase). A supernatant of homogenate in 50 mM potassium phosphate buffer at pH 7.5 was prepared by centrifuging the homogenate at 1000 g for 10 min at 4 °C. Subsequently, 900 μL of glutathione peroxidase assay buffer, 50 μL of NADPH assay reagent, and 50 μL of the sample were added to the cuvette, and the contents were mixed by inversion. The reaction was started by adding 10 μL of 30 mM *tert*-butyl hydroperoxide or 80% cumene hydroperoxide. Absorbance was recorded by the following program; wavelength, 340 nm; initial delay, 15 s; interval, 10 s; number of readings, 6. The activity of the enzyme was the sum of the values obtained using 30 mM *tert*-butyl hydroperoxide and 80% cumene hydroperoxide [34]. The level of glutathione was expressed in terms of μmol/min/mg of protein.

### 3.9. Histopathology

Stomach tissues were fixed in 10% neutral formalin and embedded in paraffin, and 4-μm-thick sections were prepared and stained with hematoxylin and eosin by standard procedures.

### 3.10. Statistical Analysis

All values were represented as the mean ± SEM. Data were analyzed by ANOVA according to the General Linear Model procedure. The means were compared by Tukey’s Studentized Range (HSD) test to detect significant differences at *p* < 0.05.

## 4. Conclusions

In the present study, we hypothesized that selenium could inhibit ethanol-induced gastric mucosal lesions, and such protective effects may directly involve its antioxidant property. Our data indicated that selenium showed gastroprotective effects against ethanol-induced gastric mucosal lesions through prevention of lipid peroxidation and activation of radical scavenging enzymes, such as SOD, catalase, and glutathione peroxidase. Thus, selenium is a potent remedy for gastric mucosal lesions and its use may offer an attractive strategy for curing gastric lesions in humans.

## References

[1] Wallace J.L., Granger D.N. (1996). The cellular and molecular basis of gastric mucosal defense. FASEB J.

[2] Laine L., Takeuchi K., Tarnawski A. (2008). Gastric mucosal defense and cytoprotection: Bench to bedside. Gastroenterology.

[3] Eastwood G.L., Kirchner J.P. (1974). Changes in the fine structure of mouse gastric epithelium produced by ethanol and urea. Gastroenterology.

[4] Brzozowski T., Konturek P.C., Konturek S.J., Brzozowska I., Pawlik T. (2005). Role of prostaglandins in gastroprotection and gastric adaptation. J. Physiol. Pharmacol.

[5] Lulu D.J., Dragstedt L.R. (1970). Massive bleeding due to acute hemorrhagic gastritis. Arch. Surg.

[6] Tarnawski A., Stachura J., Ivey K.J., Mach T., Bogdal J., Klimczyk B. (1981). Ethanol induced duodenal lesions in man. Protective effect of prostaglandin. Prostaglandins.

[7] Laine L. (1991). Endoscopic therapy for peptic ulcer hemorrhage: Heater probe and alcohol injection. Gastroenterology.

[8] Szabo S. (1987). Mechanisms of mucosal injury in the stomach and duodenum: Time-sequence analysis of morphologic, functional, biochemical and histochemical studies. Scand. J. Gastroenterol.

[9] Gottfried E.B., Korsten M.A., Lieber C.S. (1978). Alcohol-induced gastric and duodenal lesions in man. Am. J. Gastroenterol.

[10] Szabo S., Trier J.S., Brown A., Schnoor J. (1985). Early vascular injury and increased vascular permeability in gastric mucosal injury caused by ethanol in the rat. Gastroenterology.

[11] Lutnicki K., Wrobel J., Ledwozyw A., Trebas-Pietras E. (1992). The effect of calcium ions on the intensity of peroxidation processes and the severity of ethanol-induced injury to the rat gastric mucosa. Arch. Vet. Pol.

[12] Park J.G., Oh G.T. (2011). The role of peroxidases in the pathogenesis of atherosclerosis. BMB Rep.

[13] Pihan G., Regillo C., Szabo S. (1987). Free radicals and lipid peroxidation in ethanol- or aspirin-induced gastric mucosal injury. Dig. Dis. Sci.

[14] Szelenyi I., Brune K. (1988). Possible role of oxygen free radicals in ethanol-induced gastric mucosal damage in rats. Dig. Dis. Sci.

[15] Shaw S., Herbert V., Colman N., Jayatilleke E. (1990). Effect of ethanol-generated free radicals on gastric intrinsic factor and glutathione. Alcohol.

[16] Ito M., Suzuki Y., Ishihara M., Suzuki Y. (1998). Anti-ulcer effects of antioxidants: Effect of probucol. Eur. J. Pharmacol.

[17] Alvarez-Suarez J.M., Dekanski D., Ristić S., Radonjić N.V., Petronijević N.D., Giampieri F., Astolfi P., González-Paramás A.M., Santos-Buelga C., Tulipani S. (2011). Strawberry polyphenols attenuate ethanol-induced gastric lesions in rats by activation of antioxidant enzymes and attenuation of MDA increase. PLoS One.

[18] Ibrahim I.A., Qader S.W., Abdulla M.A., Nimir A.R., Abdelwahab S.I., Al-Bayaty F.H. (2012). Effects of *Pithecellobium jiringa* ethanol extract against ethanol-induced gastric mucosal injuries in Sprague-Dawley rats. Molecules.

[19] Mei X., Xu D., Xu S., Zheng Y., Xu S. (2012). Novel role of Zn(II)-curcumin in enhancing cell proliferation and adjusting proinflammatory cytokine-mediated oxidative damage of ethanol-induced acute gastric ulcers. Chem. Biol. Interact.

[20] Rotruck J.T., Pope A.L., Ganther H.E., Swanson A.B., Hafeman D.G., Hoekstra W.G. (1973). Selenium: Biochemical role as a component of glutathione peroxidase. Science.

[21] Marcocci L., Floche L., Packer L. (1997). Evidence for a functional role of the selenocysteine residue in mammalian thioredoxin reductase. Biofactors.

[22] Lee S.R., Bar-Noy S., Kwon S., Levine R.L., Stadtman T.C., Rhee S.G. (2000). Mammalian thioredoxin reductase: Oxidation of the *C*-terminal cysteine/selenocysteine active site forms a thioselenide, and replacement of selenium with sulfur markedly reduces catalytic activity. Proc. Natl. Acad. Sci. USA.

[23] Imam S.Z., Newport G.D., Islam F., Slikker W., Ali S.F. (1999). Selenium, an antioxidant, protects against methamphetamine-induced dopaminergic neurotoxicity. Brain Res.

[24] Halliwell B., Gutteridge J.M. (1992). Biologically relevant metal ion-dependent hydroxyl radical generation: An update. Fed. Eur. Biochem. Soc. Lett.

[25] Sanmartin C., Plano D., Font M., Palop J.A. (2011). Selenium and clinical trials: New therapeutic evidence for multiple diseases. Curr. Med. Chem.

[26] Loef M., Schrauzer G.N., Walach H. (2011). Selenium and Alzheimer’s disease: A systematic review. J. Alzheimers Dis.

[27] Hogeboom G.H., Colowick S.P., Kaplan N.O. (1955). Methods in Enzymology.

[28] Bradford M.M. (1976). A rapid and sensitive method for the quantitation of microgram quantities of protein utilizing the principle of protein-dye binding. Anal. Biochem.

[29] Mihara M., Uchiyama M. (1978). Determination of malonaldehyde precursor in tissues by thiobarbituric acid test. Anal. Biochem.

[30] Ohkawa H., Ohishi N., Yagi K. (1979). Assay for lipid peroxides in animal tissues by thiobarbituric acid reaction. Anal. Biochem.

[31] McCord J.M., Fridovich I. (1967). Superoxide dismutase, an enzymatic function for erythrocuprein (hemocuprein). J. Biol. Chem.

[32] Aebi H., Bergmeyer H.U. (1974). Methods of Enzymatic Analysis.

[33] Lawrence R.A., Burk R.F. (1976). Glutathione peroxidase activity in selenium-deficient rat liver. Biochem. Biophys. Res. Commun.

[34] Kim H., Lee S.W., Baek K.M., Park J.S., Min J.H. (2011). Continuous hypoxia attenuates paraquat-induced cytotoxicity in the human A549 lung carcinoma cell line. Exp. Mol. Med.

